# Complete genome sequences of six duckweed-associated bacterial strains for studying community assembly in synthetic plant microbiome

**DOI:** 10.1128/mra.01280-23

**Published:** 2024-03-01

**Authors:** Hidehiro Ishizawa, Minami Tada, Yosuke Tashiro, Masashi Kuroda, Daisuke Inoue, Hideo Dohra, Hiroyuki Futamata, Michihiko Ike

**Affiliations:** 1 Department of Applied Chemistry, Graduate School of Engineering, University of Hyogo, Himeji, Hyogo, Japan; 2 Division of Sustainable Energy and Environmental Engineering, Graduate School of Engineering, Osaka University, Suita, Osaka, Japan; 3 Research Institute of Green Science and Technology, Shizuoka University, Suruga-ku, Shizuoka, Japan; 4 Department of Engineering, Graduate School of Integrated Science and Technology, Shizuoka University, Hamamatsu, Japan; 5 Graduate School of Science and Technology, Shizuoka University, Hamamatsu, Japan; 6 Faculty of Social and Environmental Studies, Tokoha University, Shizuoka, Japan; 7 Department of Science, Graduate School of Integrated Science and Technology, Shizuoka University, Shizuoka, Japan; The University of Arizona, Tucson, Arizona, USA

**Keywords:** plant-microbe interactions, microbial communities

## Abstract

We report the complete genome sequences of six bacterial strains isolated from a floating macrophyte, duckweed. These six strains, representing the six dominant families of the natural duckweed microbiome, establish a simple model ecosystem when inoculated onto sterilized duckweed. Their genomes would provide insights into community assembly in plant microbiome.

## ANNOUNCEMENT

Understanding the structuring principle of plant-associated microbiomes presents a significant challenge due to their intricate nature and the presence of uncultured or uncharacterized members. To address this issue, researchers have increasingly turned to the use of synthetic ecosystems, which involve a sterilized plant and isolated microbial strains (1, 2). Previously, we established a synthetic ecosystem with six bacterial strains and their original host, duckweed (*Lemna minor*, RDSC5512) (3). This synthetic ecosystem shows a remarkably stable community structure and closely mirrors the family-level structure found in natural duckweed microbiomes (3). Due to its highly deterministic nature, this unique system provides a valuable platform for studying microbial community assembly (4). This study set out to obtain the genomes of the six bacterial strains within the synthetic ecosystem, *Acidovorax* sp. DW039, *Novosphingobium olei* DW067, *Chryseobacterium gambrini* DW100, *Methylophilus* sp. DW102, *Asticcacaulis* sp. DW145, and *Herbaspirillum* sp. DW155, isolated from duckweed and belonging to dominant families of natural duckweed microbiome (3).

The genomic DNA was extracted using GenElute bacterial genomic DNA kit (Sigma-Aldrich, St. Louis, MO, USA), after culturing single colonies streaked from glycerol stock in R2A medium (supplemented with 2% methanol for DW102) at 25°C with rotary shaking (150 rpm). The genomic DNA was sheared using a g-TUBE device (Covaris, Woburn, MA, USA) with Eppendorf 5424 centrifuge (4,000 rpm, 1 min), and 8–13 kb fragments were selected using the BluePippin system with a 0.75% agarose gel cassette (Sage Science, Inc., Beverly, MA, USA) for PacBio Sequel II HiFi sequencing (Pacific Biosciences, Menlo Park, CA, USA). SMRTbell templates were prepared according to the manufacturer’s instructions and sequenced at Macrogen, Inc. (Seoul, South Korea). PacBio HiFi reads with lengths of ≥7.5  kb or 10 kb were filtered using Seqkit v. 0.8.0 (5) and assembled using Flye v. 2.8.3 (6) or HiCanu v. 2.1.1 (7), as summarized in Table 1. The resulting contigs were manually rotated using Geneious Prime 2022 (8) to place the *dnaA* gene at the first position of the circular chromosome sequence. Non-chromosomal circular contigs with replication initiation factor protein and *parAB* genes were considered plasmids. The HiFi reads were aligned with the genome sequence using pbmm2 v. 1.3.0 (https://github.com/PacificBiosciences/pbmm2), and assembly errors were corrected using Pilon v. 1.23 (9). Gene prediction and annotation were performed with DFAST-core v. 1.2.14 (10) using GeneMarkS2 v. 1.14_1.25 (11), RNAmmer v. 1.2 (12), and tRNAscan-SE v. 2.0.5 (13) to predict protein-coding sequences (CDSs), rRNAs, and tRNAs, respectively. Default parameters were used for all software unless otherwise specified. Information on the obtained reads and generated genome sequences is summarized in Table 1.

**TABLE 1:** General features of genome sequencing and assembly of six duckweed-associated bacterial strains

Category	Feature	*Acidovorax* sp. DW039	*Novosphingobium olei* DW067	*Chryseobacterium gambrini* DW100	*Methylophilus* sp*.* DW102	*Asticcacaulis* sp. DW145	*Herbaspirillum* sp*.* DW155
HiFi reads	No. of reads	101,183	89,716	40,726	48,124	38,188	113,053
	Total bases (bp)	1,068,823,671	916,674,061	439,519,806	553,285,216	394,957,226	1,213,162,625
	N50 (bp)	10,706	10,486	11,019	11,808	10,548	10,864
Filtered reads	Filtered length	10,000	7,500	10,000	7,500	7,500	7,500
	No. of reads	62,908	89,495	27,421	48,044	38,021	112,745
	Total bases (bp)	716,099,019	915,493,911	316,912,958	552,999,199	394,171,012	1,212,239,427
	N50 (bp)	11,408	10,488	11,642	11,810	10,552	10,866
	Mean coverage (×)	137.19	229.67	69.02	183.28	92.67	221.42
Assembly	No. of replicons	2	1	1	1	4	1
	Genome size (bp)	5,219,685	3,986,061	4,591,558	3,017,312	4,253,521	5,474,944
	Chromosome 1 (bp)	4,854,771	–	–	–	2,475,838	-
	Chromosome 2 (bp)	–	–	–	–	1,342,519	-
	Plasmid 1 (bp)	364,914	–	–	–	344,984	-
	Plasmid 2 (bp)	–	–	–	–	90,180	-
	GC content (%)	62.12	65.43	64.06	51.41	60.36	62.25
	No. of CDSs	4,610	3,720	4,074	2,804	3,708	4,933
	No. of rRNAs	12	6	18	9	9	9
	No. of tRNAs	54	53	90	46	52	65
	Tools	Flye 2.8.3 Pilon 1.23	Flye 2.8.3 Pilon 1.23	HiCanu 2.1.1 Pilon 1.23	Flye 2.8.3 Pilon 1.23	Flye 2.8.3 Pilon 1.23	Flye 2.8.3 Pilon 1.23
	Genome size setting	5.2 M	4 M	4.6 M	3 M	4.2 M	5.5 M
Accession data	GenBank accession no.	AP029019–AP029020	AP029021	AP029022	AP029023	AP029024–AP029027	AP029028
	SRA accession no.	DRR514962	DRR514963	DRR514964	DRR514965	DRR514966	DRR514967

The genome assembly for each strain yielded one to two closed chromosomes, ranging in size from 1,342,519 to 5,474,944 bp, accompanied by up to two closed plasmids per strain (ranging from 90,180 to 364,914 bp) (Table 1). These genomes contained putative genes associated with plant colonization (e.g., flagellar assembly, chemotaxis, and catabolism of aromatic compounds). Further genome analysis will help elucidate how these six bacterial strains establish and maintain stable coexistence on the plant surfaces.

## Data Availability

The genome sequences of the six bacterial strains have been deposited at DDBJ/ENA/GenBank under accession no. AP029019–AP029028. They are linked to BioProject accession no. PRJDB17029, Biosample accession no. SAMD00659498–SAMD00659503, and DDBJ Sequence Read Archive accession no. DRA017487.
